# Overexpressing *BrWRKY22* Delays Flowering and Leaf Senescence via Inhibition of GA Biosynthesis in *Brassica rapa*

**DOI:** 10.3390/plants14111658

**Published:** 2025-05-29

**Authors:** Junaite Bin Gias Uddin, Tingzhen Zhuo, Xiaojie Li, Xuan Wu, Zhuoyu Wu, Habiba, Yujun Ren, Ying Miao

**Affiliations:** Fujian Provincial Key Laboratory of Plant Functional Biology, College of Life Sciences, Fujian Agriculture & Forestry University, Fuzhou 350002, China; junaiteturjo@outlook.com (J.B.G.U.); zhuotingzhen@163.com (T.Z.); 12305014069@fafu.edu.cn (X.L.); 12405014033@fafu.edu.cn (X.W.); 12305014039@fafu.edu.cn (Z.W.); habiba09248@gmail.com (H.)

**Keywords:** chlorophyll, gibberellin, WRKY transcription factor, flowering, senescence, *Brassica*

## Abstract

WRKY transcription factors play a predominant role in plant stress responses, as well as growth and development. Although *WRKY* genes have been extensively studied in model plants, little is known about them in *Brassica rapa*. In this study, the *BrWRKY22* gene was isolated and characterized. BrWRKY22 is nuclear localized and has self-activation and dimerization activity. *BrWRKY22* was highly expressed in young leaves, roots, and stems. The overexpressed *BrWRKY22 Arabidopsis* and *Brassica rapa* lines exhibited a dwarfish, delayed flowering and leaf senescence phenotype compared to the wild-type (WT). Molecular evidence showed that the transcript levels of *BrCHLP* are increased, whereas those of *BrLFY*, *BrSOC1*, *BrGA20OX2*, *BrGA3OX1*, and *BrGASA6* are significantly decreased in *BrWRKY22* overexpressing plants compared to the WT. BrWRKY22 can bind directly to the promoters of *BrCHLP* and *BrGA20OX2*, activating *BrCHLP* and repressing *BrGA20OX2* gene transcription. The chlorophyll b and tocopherol levels are increased, whereas the GA and ABA levels are significantly decreased, in three-week-old *BrWRKY22* overexpressing *Brassica* lines compared to the WT. Collectively, our results suggest that BrWRKY22 directly controls chlorophyll b and GA biosynthesis and plays a repressive role in leaf senescence and the initiation of flowering in *Brassica rapa* plant development.

## 1. Introduction

As one of the most important oil crops, rapeseed (*Brassica rapa var. Chinensis*) has many industrial and everyday uses. It not only provides an edible oil for humans, but it can also be used in animal feed and as an ornamental flower [1]. Leaf senescence and flowering time are important agronomic traits for crop production. Whether leaf senescence and flowering occur at the right time has a direct effect on plant reproduction and yield [2]. In cereals, early leaf senescence and flowering can prolong the grain filling phase and help increase the seed yield potential [3].

Flowering time and leaf senescence are tightly regulated during monocarpic senescence. In annual plants, it is widely recognized that monocarpic senescence and eventual death are closely linked to leaf senescence, flowering, and seed maturation [4,5]. Research has shown that premature leaf senescence often coincides with early flowering, whereas delayed leaf senescence does not always result in delayed flowering [5,6,7]. A natural process of leaf senescence has been documented in the late-flowering constants mutant, indicating that leaf senescence can occur independently from the onset of flowering [8]. While the mechanisms underlying flowering time and leaf senescence during monocarpic senescence have been extensively studied individually, the transcription factors (TFs) that coordinate flowering and leaf senescence remain largely unidentified.

Phytohormones play a crucial role in the regulation of leaf senescence and flowering. For example, brassinosteroids (BRs), jasmonic acid (JA), ethylene, salicylic acid (SA), and abscisic acid (ABA) have shown to accelerate leaf senescence, whereas cytokinins (CTKs), gibberellins (GAs), and auxins function in the opposite direction for leaf senescence and flowering [9]. GAs are a large family of tetracyclic diterpenes that are widely involved in various growth and developmental processes throughout the plant life cycle [10,11]. GAs have been shown to inhibit leaf senescence across plant species [12]. It is widely accepted that GAs are negative regulators of leaf senescence and flowering [9,13]. It has been reported that the antisense inhibition of GIGANTEA (BoGI) in kale (*Brassica oleracea* var. sabellica) delayed both leaf senescence and flowering [14].

WRKY TFs are plant-specific proteins and constitute one of the largest TF families in plants [15]. They are involved in many physiological and biochemical processes, such as disease resistance [16,17,18], development [19,20], metabolism [21,22], hormone signal transduction [18,23], and biotic and abiotic stress responses [24,25], In *Arabidopsis*, several WRKY proteins were found to be involved in the regulation of leaf senescence and flowering time. For example, AtWRKY71 can activate the expression of flowering genes and accelerate the leaf senescence and flowering process in *Arabidopsis thaliana* [26]. AtWRKY12 and AtWRKY13 oppositely modulate the flowering time under short day conditions [27], while AtWRKY75 functions as a positive regulator of leaf senescence and flowering in *Arabidopsis* [28]. AtWRKY1 is a crucial TF that regulates monocarpic senescence in *Arabidopsis*. It promotes flowering by directly repressing *Flowering Locus C* (*FLC)* expression and induces leaf senescence by activating SA biosynthesis genes. It also directly activates the genes involved in nitrogen assimilation and transport for remobilization from senescing leaves to seeds [29]. In addition, AtWRKY22, a dark-induced WRKY member, plays a role in accelerating leaf senescence and early flowering under dark-induced conditions [30]. In *Brassica* crops, WRKY genes were identified in *Brassica rapa* and *Brassica oleracea*: 145 and 142, respectively [31,32]. Some studies revealed that a transcriptional regulator, BrWRKY6, was associated with gibberellin-suppressed leaf senescence of Chinese flowering cabbage [33,34]. The genetics background of days to flowering, maturity, and plant height in natural and derived forms of *Brassica rapa* have been investigated [35]. However, the function of the WRKY gene family in regulating leaf senescence and flowering in *Brassica* is still limited.

In the present study, a *BrWRKY22* gene (Bra037368) was isolated and characterized from *Brassica rapa*. We performed a preliminary analysis of the gene structure, evolutionary relationships, and expression patterns of *BrWRKY22*. The overexpressed *BrWRKY22* gene in *Arabidopsis* and *Brassica rapa* plants was constructed and phenotyped. Furthermore, Reverse transcription quantitative PCR (RT-qPCR) analysis was used to detect the alteration of the transcript level of geranylgeraniol reductase gene (*BrCHLP)*, *LEAFY (BrLFY)*, suppressor of overexpression of CO1 (*BrSOC1)*, gibberellin 20 oxidase 2 (*BrGA20OX2)*, gibberellin 3 ocidase 1 (*BrGA3OX1)*, and *gibberellic acid-stimulated Arabidopsis* 6 (*BrGASA6)* in overexpressing *BrWRKY22* plants compared to the wild type (WT) and used the yeast one-hybrid and dual-luciferase assays to investigate whether BrWRKY22 directly controls chlorophyll biosynthetic enzyme *BrCHLP* expression and active GA biosynthetic enzyme *BrGA20OX2* expression. Furthermore, we measured the chlorophyll and hormones GA and ABA levels in the *BrWRKY22* overexpression lines compared to the WT using high performance liquid chromatography (HPLC).

## 2. Results

### 2.1. Sequence Alignment and Phylogenetic Relationship of the BrWRKY22 Protein

**Figure 1:**
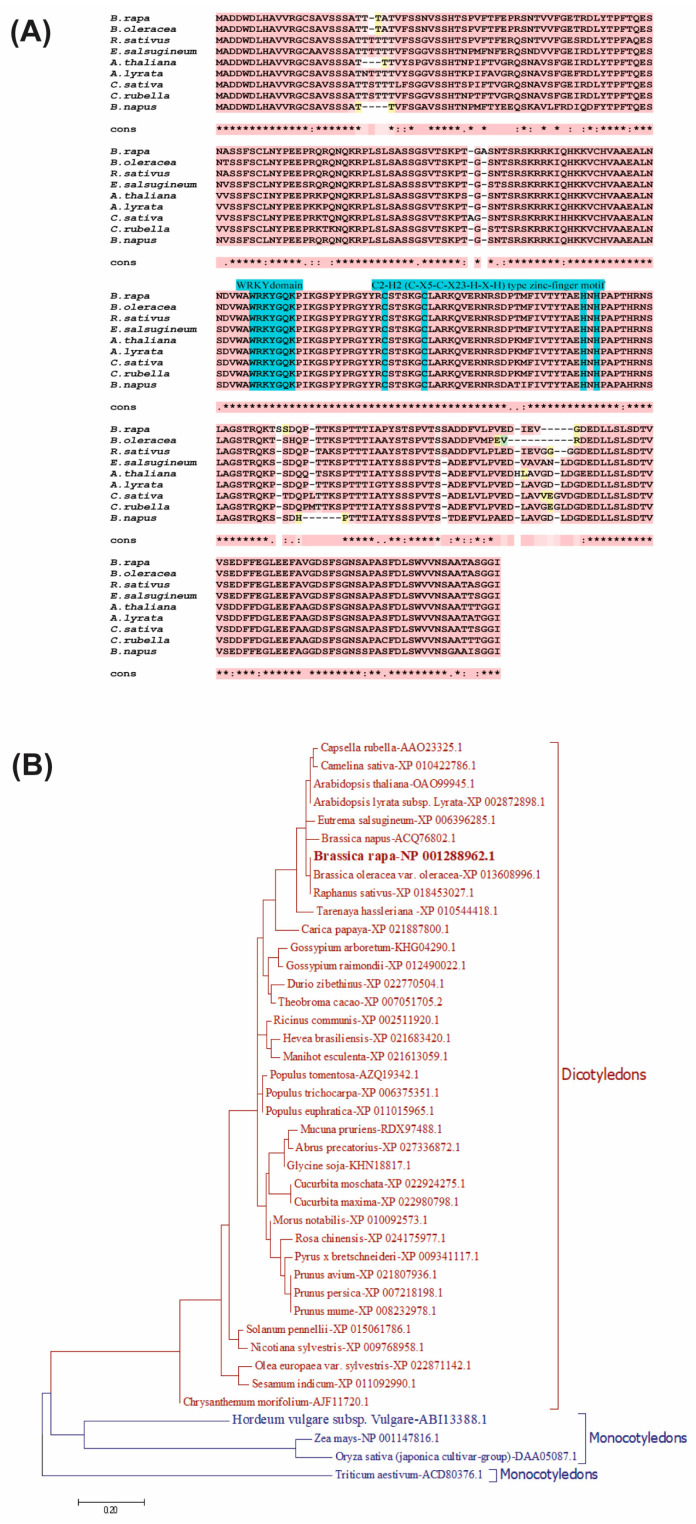
Detection of conserved sequences and evolutionary relationship of the WRKY22 protein. (**A**) Multiple sequence alignment of the WRKY22 protein in *B. rapa*, *B. oleracea*, *R. sativus*, *E. salsugineum*, *A. thaliana*, *A. lyrata*, *C. sativa*, *C. rubella*, and *B. napus*. pink color and * indicate conserve sequences; pink color and : indicate mostly conserve sequences; pink color and . indicate partially conserve sequences; blue color, WRKY domain and C2H2 zinc finger motif; yellow color, different region. (**B**) Phylogenetic relationship of the WRKY22 proteins in various dicots and monocots. The phylogenetic tree was constructed using the maximum likelihood method with MEGA 7 software. pink color, dicotyledons; blue color, monocotyledons.

The coding sequence of *BrWRKY22* encodes a protein of 298 amino acid residues that contains a conserved WRKY domain at its C-terminal end, along with a C2H2 (C-X5-C-X23-H-X-H)-type zinc finger-like motif (Figure 1A). Multiple sequence alignment of the WRKY22 proteins showed the same WRKY domain with a highly conserved WRKYGQK motif and C2H2 (C-X5-C-X23-H-X-H)-type zinc finger-like motif. Therefore, based on the sequence characteristics, it was found that BrWRKY22 also belongs to Group 2E of the WRKY superfamily.

High-sequence similarity might depict similar functions. Therefore, we aligned amino acid sequences of various dicots and monocots, and evolutionary analysis was performed. Further phylogenetic analysis of the WRKY22 members showed that monocots and dicots tend to cluster separately (Figure 1B). *BrWRKY22* showed higher similarity with the *WRKY22* sequences of *B. oleracea*, *R. sativus*, *E. salsugineum*, *A. thaliana*, *A. lyrata*, *C. sativa*, *C. rubella*, and *B. napus*. This result was consistent with the results of the amino acid alignment analysis.

To better understand the *BrWRKY22* gene structure, we performed a gene sequence analysis. It was found that the *BrWRKY22* gene comprised a total length of 1.7 kb, including two untranslated regions (UTR), three exons, and two introns. The *BrWRKY22* exons are 444 bp, 110 bp, and 340 bp long, and the introns are situated in between these three exons (Supplementary Figure S1).

### 2.2. BrWRKY22 Localizes to the Nucleus and Forms a Homomeric Oligomer

To investigate the subcellular localization, initially, we adopted a bioinformatics approach for the prediction of protein location. Subcellular localization prediction by UniProtKB, Plant-mPLoc, LOCALIZER, WoLF PSORT, and BaCelLo identified that the BrWRKY22 protein is localized in the nucleus.

**Figure 2:**
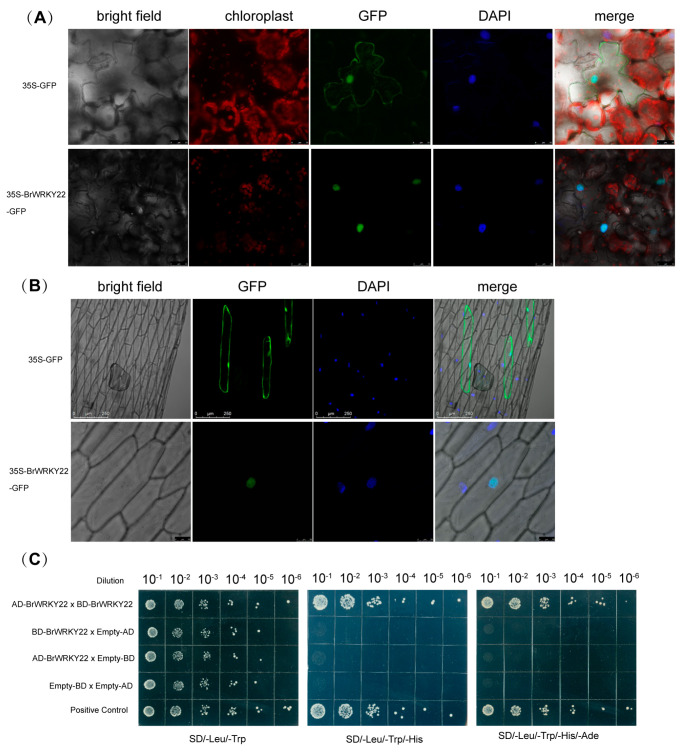
Subcellular localization and transactivation of BrWRKY22. (**A**) Confocal microscopy showing GFP expression of 35S-BrWRKY22:GFP in *Nicotiana benthamiana* leaves. (**B**) GFP imaging in onion epidermal cells using a 35S-BrWRKY22:GFP construct. (**C**) Empty pGADT7 × Empty pGBKT7 was used as the negative control, and pGADT7-AtHAD15× pGBKT7-AtHDA15 was used as the positive control. The transformed yeast cells were streaked on SD/-Leu/-Trp, SD/-Leu/-Trp/-His, and SD/-Leu/-Trp/-His/-Ade. Apart from the positive controls, only pGADT7-BrWRKY22× pGBKT7-BrWRKY22 grows colonies in SD/-Leu/-Trp/-His and SD/-Leu/-Trp/-His/-Ade mediums. To check whether BrWRKY22 can interact with itself when it is transcribed or forms a dimer in the nucleus when it is functionally activated, BrWRKY22 and BrWRKY22 were fused with the Gal4 DNA-binding domain (in the pBD bait vector) and activation domain (in the pAD prey vector). With the co-transformation of AD-BrWRKY22 and BD-BrWRKY22 to AH109 yeast cells, the homomeric oligomer of BrWRKY22 was detected by the growth of yeast cells on the selection media deficient in Leu, Trp, His, and Ade (**C**). When empty prey and bait vectors were transformed with AD-BrWRKY22 and BD-BrWRKY22, the colonies were not grown in the selected medium, whereas the positive control grew well, suggesting that BrWRKY22 can form a homomeric oligomer in yeast cells. These results indicate that BrWRKY22 can physically interact with BrWRKY22, forming a homomeric oligomer.

To further confirm the subcellular localization of BrWRKY22, we generated the vector comprising the BrWRKY22 coding sequence fused with GFP, under the control of the *35S CaMV* promoter. A fusion construct, along with positive control (pPZP212-GFP-HA), was transiently introduced into onion epidermal cells and *Nicotiana benthamiana* leaves. Confocal microscopy confirmed that BrWRKY22 is nuclear-localized in both transiently induced onion epidermal cells (Figure 2A) and *N. benthamiana* leaves (Figure 2B), in contrast to the positive control, which was found to be expressed throughout the cell, including the plasma membrane, cytosol, and the nuclei.

### 2.3. Expression Analysis of BrWRKY22 in Different Tissues

**Figure 3:**
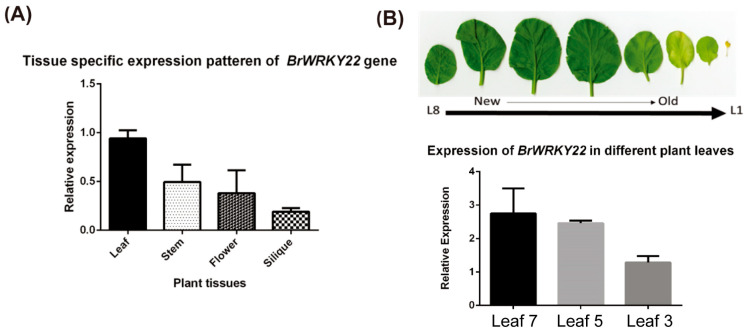
Expression profile of *BrWRKY22*. (**A**) Tissue-specific expression patterns of *BrWRKY22* showing in comparison to other tissues, with the *BrWRKY22* highest expression observed in leaf tissue. (**B**) Senescence symptoms of detached leaves and qPCR analysis showing that the *BrWRKY22* gene is most expressed in young leaves of *Brassica rapa*. In this experiment, three biological replicates were tested, and each biological replicate contained leaves from three independent plants.

To further characterize BrWRKY22, we examined their expression in *Brassica rapa* using RT-qPCR. First, we examined the transcript levels of *BrWRKY22* genes in different *Brassicas rapa* tissues. As shown in Figure 3A, the expression of *BrWRKY22* gene was detected in all tissues examined, but the expression levels in the leaves, stem, and flower were substantially higher. Then, to evaluate the expression pattern of *BrWRKY22* during different leaf stages, RT-qPCR was performed using *Brassica rapa* leaves of different ages. The expression of the genes was highest at a young age but steadily decreased with age. In the 7th leaf stage, the expression levels were about 1.6 times higher than the 3rd leaf stage (Figure 3B). Thus, the expression of *BrWRKY22* in leaves started at moderate levels, increased steadily in expanding leaves, and remained highly expressed in the young, expanded leaves but gradually decreased in the old leaves. This result suggests that expression of the genes in *Brassica rapa* leaves is developmentally regulated and related to senescence.

### 2.4. BrWRKY22 Overexpressed Transgenic Lines in Arabidopsis and in Brassica rapa Exhibit a Delay in Flowering Time and Leaf Senescence

To explore the role of BrWRKY22, we generated *Arabidopsis* transgenic lines harboring *pCBIM-Bra037368(BrWRKY22)-Flag* plasmid. The developed transgenic plants were screened for two generations on hygromycin-resistant plates (Supplementary Figure S2). After that, to better analyze the phenotypic expression of *BrWRKY22* in overexpressed *Arabidopsis* transgenic lines, we confirmed the successful integration and expression of the transgene. The total protein from the leaves of *BrWRKY22* overexpressed transgenic lines, as well as wild-type plants, was extracted, and the expression was analyzed through Western blotting (Supplementary Figure S3). The *BrWRKY22* gene’s (harboring *FLAG: pCBIM-Bra037368-Flag*) successful transformation into *Arabidopsis* transgenic lines was confirmed with the FLAG antibody. Among the expected overexpression lines, 28-1, 28-2, and 28-10 showed higher protein expression, depicting successful transformation and significant expression.

**Figure 4:**
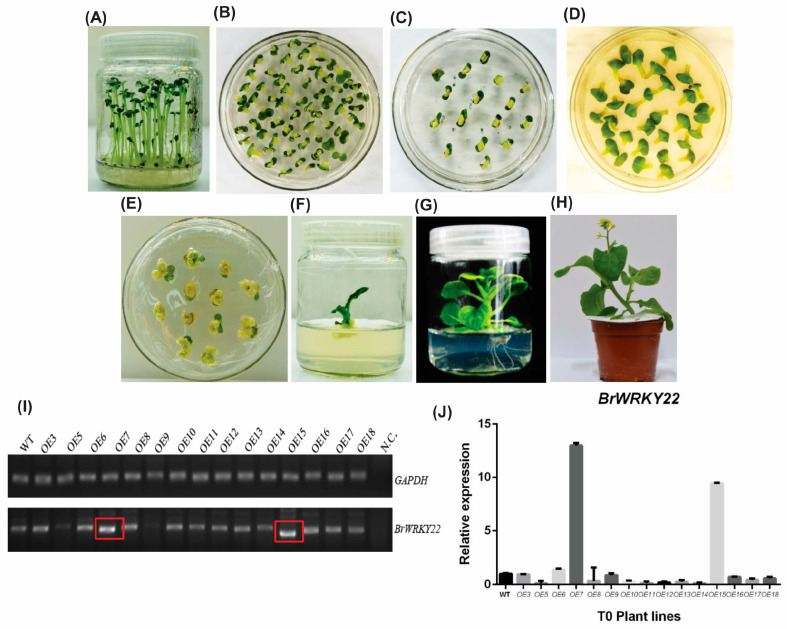
*Agrobacterium*-mediated transformation of the OE vector into *Brassica rapa*. (**A**) The growth of *Brassica rapa* seeds in the planting medium after 5 days. (**B**) The growth of explants (cotyledons) after 3 days in the pre-culture medium. (**C**) The growth of *Agrobacterium*-treated explants in the co-culture medium after 2 days. (**D**) The growth of explants in the recovery medium 4 days after transferring. (**E**) Callus induction from explants, full grown callus, and plant induction (red circled) from callus in the screening medium. (**F**) The growth of the plant in the shooting medium. (**G**) Growth of roots in the rooting medium. (**H**) Transferring a plant from the rooting medium to water for hardening. (**I**) After 7 days of hardening, the plants were transferred into soil. (**I**) Semi-quantitative PCR confirmation of the OE BrWRKY22 lines. RO water was used as the negative control (N.C.). Red box shows the highest amount band. (**J**) Quantitative PCR analysis showing overexpression of the *BrWRKY22* gene in OE7 and OE15.

To further investigate the role of BrWRKY22, we generated *Brassica rapa* overexpressing lines by the *Agrobacterium*-mediated transformation of the *pPZP212-BrWRKY22-Flag* plasmid (Figure 4A–H). Then, we performed reverse transcription PCR (RT-qPCR), which showed that all the developed transgenics showed a successful integration of the transgene into the genome of *Brassica rapa* (Figure 4I). Furthermore, real-time PCR (RT-PCR) depicted that the *OE7* and *OE15* transgenic lines have significant expression patterns, as compared to the wild-types (WT) (Figure 4J).

**Figure 5:**
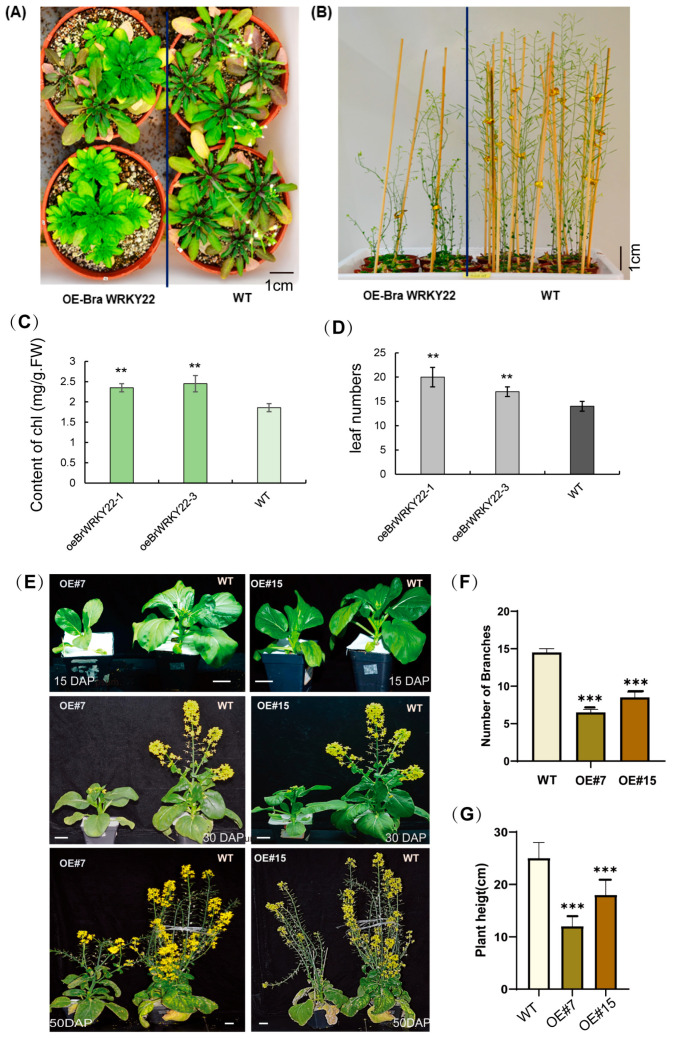
Phenotypic analysis of OE BrWRKY22 in the *Arabidopsis thaliana* and in *Brassica rapa* transgenic lines. Transgenic *Arabidopsis* plants overexpressing BrWRKY22 were identified by Western blotting (Supplementary Figure S3). Homozygous T3 progeny of transgenic BrWRKY22 (lines 28-1 and 28-2) were grown in a 12/12-h light/dark photoperiod. Images of representative plants were taken at 6 (**A**) and 8 (**B**) weeks after germination. (**A**) Leaf and flower time comparisons of the WT and OE BrWRKY22 in *Arabidopsis* plant lines. (**B**) *BrWRKY22* overexpressed *Arabidopsis* plants and wild-type plants showing stunted and delayed flowering plant growth in OE lines. (**C**) The contents of chlorophyll of OE BrWRKY22 in *Arabidopsis* plant lines relative to the WT. (**D**) The number of rosette leaves of OE BrWRKY22 in *Arabidopsis* plant lines relative to the WT. (**E**) Overexpression of *BrWRKY22* via *Agrobacterium*-mediated transformation in lines OE#7 and OE#15 decreases the plant height and flowering time in *Brassica rapa*. Scale bar = 1 cm. (**F**) Average number of branches in the WT and OE#7 and OE#15 (**G**) Average plant height in the WT and OE#7 and OE#15. DAP indicates day after planting. The bars represent the means ± SEs from five plants of each line. Independent *t*-tests revealed highly significant (** *p* < 0.01, *** *p* < 0.001) differences between the WT and overexpressed plants.

To analyze the phenotypic expression, T2 homozygous *BrWRKY22* overexpressed *Arabidopsis* plants were observed for six weeks after planting. The leaves of six-week-old transgenic lines showed a stay green and delaying flowering phenotype, accompanied by a higher chlorophyll content and a higher number of rosette leaves in *BrWRKY22* overexpressed *Arabidopsis*, as compared to the wild-type (Figure 5A,B), indicating that BrWRKY22 might have a potential role in delaying leaf senescence and flowering. The transgenic *35S:BrWRKY22 Arabidopsis* plants showed a stunted growth with curved stems and exhibited serious morphological and developmental retardant during the entire vegetative growth phases. Moreover, all the independent lines showed a delayed flowering phenotype compared to the WT (Figure 5B). These results indicated that overexpression of *BrWRKY22* is deleterious for normal plant development in *Arabidopsis*.

To further examine the roles of BrWRKY22 in *Brassica rapa*, overexpression lines of BrWRKY22 *Brassica rapa* were selected for further investigation. RT-qPCR depicted that OE#7 and OE#15 represented the higher expression patterns of *BrWRKY22* and were selected for functional analysis. We observed that the flower buds of the transgenic plants emerged later than those of the wild-type plants under natural conditions. At 15 d after transplantation, flower buds were found on most of the wild-type plants, while this did not occur in the OE#7 and OE#15 plants (Figure 5C). Moreover, 30 days after transplantation, the wild-type plants entered the visible flower color stage, while the OE#7 and OE#15 plants were still at the flower bud development stage (Figure 5C) transgenic lines. These results indicate that BrWRKY22 can regulate flowering time in *Brassica rapa*. Furthermore, overexpressed BrWRKY22 plants exhibited a relatively lower plant height phenotype and branch number than the control plants, and the lower plant height phenotype and branch number were statistically analyzed. It was noticed that the branch number and plant height were both reduced by approximately half in the transgenic lines as compared to the WT (Figure 5D,E). Overall, these results proposed that BrWRKY22 has a converse function in flowering time regulation and plant growth control.

### 2.5. BrWRKY22 Directly Represses BrGA20OX2 Transcription and Activates BrCHLP Transcription in the Chlorophyll and GA Biosynthesis Pathway

**Figure 6:**
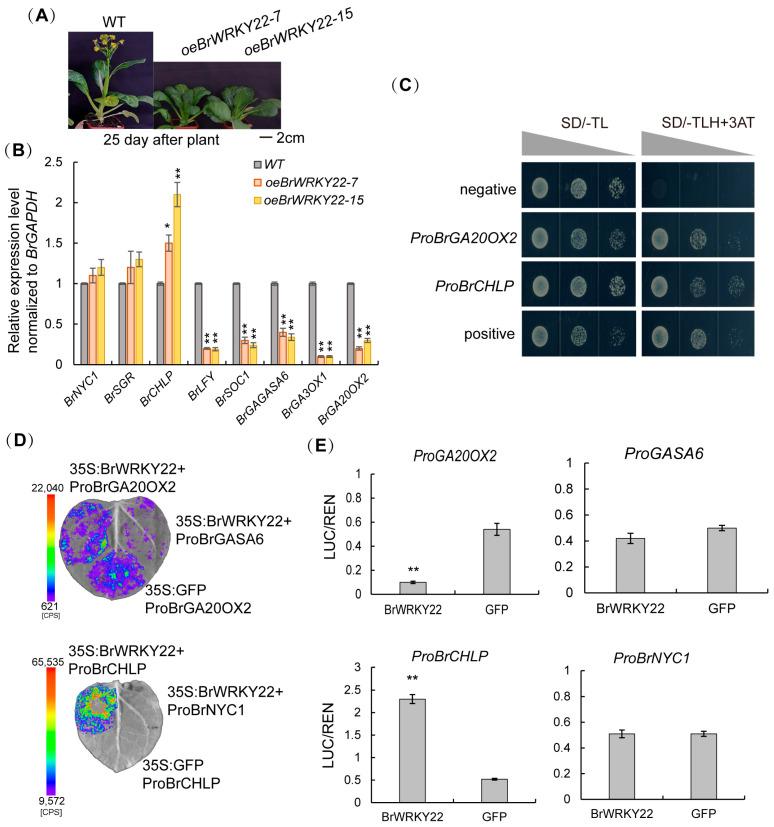
Overexpressing *BrWRKY22* directly represses *BrGA20OX2* transcription and activates *BrCHLP* transcription in the chlorophyll and GA biosynthesis pathway. (**A**) Twenty-five-DAP (days after planting)-old plants. (**B**) The expression level of genes related to chlorophyll and GA biosynthesis, as well as related to flowering in overexpressing *BrWRKY22 Brassica rapa* plants compared to the WT. Relative expression levels of the target genes were calculated using the 2^−ΔΔCt^ method. The bars represent the means ± SEs from three independent repeats. Independent *t*-tests revealed highly significant (* *p* < 0.05, ** *p* < 0.01) differences between the WT and overexpressed plants. (**C**) Yeast one-hybrid assay results. The promoter fragments of *BrGA20OX2* and *BrCHLP* were inserted into the pHIS2 expression vector. The various dilutions of colonies in the selected medium showed the activation of expression by BrWRKY22 of the *HIS* reporter gene driven by the indicated fragments in yeast. The plasmid *GAD-BrWRKY22* with empty *HIS2* plasmid was co-transformed into the yeast strain AH109 as the negative controls: P, positive; N, negative. (**D**,**E**) Luciferase fluorescent image (**D**) and activity (**E**) of co-transformed tobacco leaves with *oeBrWRKY22-GFP* and *ProBrGA20OX2:LUC*, *ProBrCHLP:LUC*, *ProBrGASA6:LUC*, and *ProBrNYC1:LUC*. oeGFP alone was used as the negative control. Shown are the mean and SE of six biological replicates. Asterisks denote statistically significant differences from the GFP-alone empty vector, calculated using the Student’s *t*-test: ** *p* < 0.01.

Since the overexpressing BrWRKY22 plants exhibited a dwarfish and delaying leaf senescence and flowering phenotype, referring to the previous research, the gene expression profiles of chlorophyll biosynthetic genes such as chlorophyll b reductase (*NYC1*), chlorophyll-degrading Mg^2+^-dechelatase (*SGR*), and *CHLP*; GA metabolic genes and signaling genes such as *GA20OX2*, *GA3OX1*, and *GASA6* [36]; and flowering-related gene such as *LFY* and *SOC1* [13] were measured by RT-qPCR in 4 week-old overexpressing *BrWRKY22* lines relative to the wild-type. The transcript level of *BrCHLP* showed a significant increase up to 1.5–2.5-fold in two overexpressing *BrWRKY22* leaves relative to the WT, while that of *BrLFY* and *BrSOC1*, as well as *BrGA2OX2*, *BrGA3OX1*, and *BrGASA6*, significantly declined 10-fold (Figure 6A,B). It supposed that BrWRKY22 is competitively involved in the chlorophyll and GA biosynthesis pathway.

The motif analysis revealed the presence of the W-box (T/CTGACC/T) cis-element in the promoters of *BrGA3OX1*, *BrGA20OX2*, *BrNYC1*, and *BrCHLP*. To investigate the regulation of BrWRKY22 on them, yeast one-hybrid and dual-luciferase assays were performed. The results of yeast one-hybrid showed that, when co-transforming *AD-BrWRKY22* effector plasmid and *BD-ProBrGA20OX2* and *BD-ProBrCHLP* reporter plasmid, the colonies are grown in the medium-deficient Trp, Leu, and His, plus 50 mM 3AT, indicating that BrWRKY22 is directly bound to the promoters of *BrGA20OX2* and *BrCHLP* in yeast cells (Figure 6C). Furthermore, the luciferase activity analysis revealed that the luciferase activity was promoted in the tobacco leaves co-transformed with both *35S:BrWRKY22-GFP* and *ProBrCHLP:LUC* plasmids, whereas that was suppressed in the tobacco leaves co-transformed with both *35S:BrWRKY22-GFP* and *ProBrGA20OX2:LUC* plasmids, while that did not alter the *ProBrNYC1:LUC* and *ProBrGASA6:LUC* plasmids compared to the co-transformed with *35S:GFP* alone with *promoter:LUC* plasmids. The results revealed that BrWRKY22 was directly bound to the promoters of *BrCHLP* and *BrGA20OX2* and activated *BrCHLP* expression but repressed *BrGA20OX2* expression (Figure 6D–F). The above results indicated that BrWRKY22 acted as a negative regulator in GA biosynthesis by directly repressing the transcription of *BrGA20OX2* and as a positive regulator in chlorophyll b synthesis by directly activating the transcription of *BrCHLP*.

### 2.6. Overexpressing BrWRKY22 Plants Decreased the GA Content but Increased the Chlorophyll b and Tocopherol Contents

**Figure 7:**
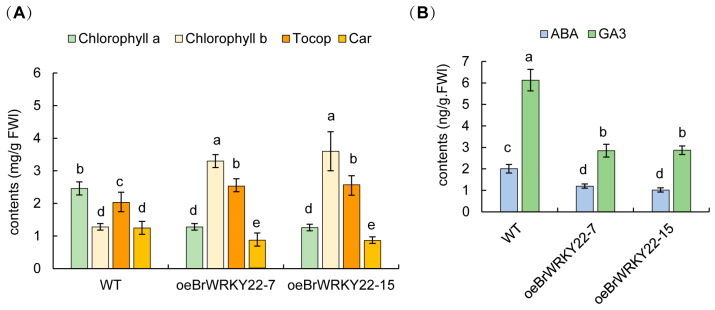
The contents of chlorophyll, tocopherol, and ABA and GA in overexpressing BrWRKY22 *Brassica rapa* leaves compared to the WT. (**A**) The contents of chlorophyll and tocopherol. (**B**) The contents of ABA and GAs. The bars represent the means ± SEs from three independent repeats. Multiple factor comparisons use the Turkey test program, and the different letters indicate significant differences; *p* < 0.05.

Since BrWRKY22 affect *BrCHLP* gene expression, it should change the chlorophyll and tocopherol contents. To this end, the amount of chlorophyll a and b and tocopherol are measured by HPLC. The results show that the contents of chlorophyll a and carotenoid are significantly decreased but those of chlorophyll b and tocopherol are significantly increased in the overexpressing *BrWRKY22* lines compared to the WT (Figure 7A). It suggests that BrWRKY22 may regulate *BrCHLP* gene expression and is involved in chlorophyll b and tocopherol biosynthesis and chlorophyll a and carotenoid competition, displaying a stay green leaf phenotype.

In the GA metabolism pathway, GA20 oxidases are located between GA12 and GA53 or GA12 and GA15 to produce GA1/GA3 or GA4, respectively, in *Arabidopsis* [37] to clarify whether BrWRKY22 affects GA20 oxidases (BrGA20OX2 and Bra022565) and GA3 oxidases (BrGA3OX1 and Bra008480) to produce the bioactive GA3 and/or GA4. The amount of GA3 and GA4 measured by HPLC shows that the contents of GA3 are significantly decreased in the overexpressing *BrWRKY22* lines compared to the WT. The GA4 content is undetectable in leaves, which is similar to *Arabidopsis*. The ABA content also declined in the overexpressing *BrWRKY22* lines (Figure 7B). It suggests that overexpressing *BrWRKY22* directly activates the *BrGA20OX2* expression and affects the levels of GA3 and ABA in *Brassica rapa* leaves, showing a dwarfish and delay flowering phenotype.

## 3. Discussion

**Figure 8:**
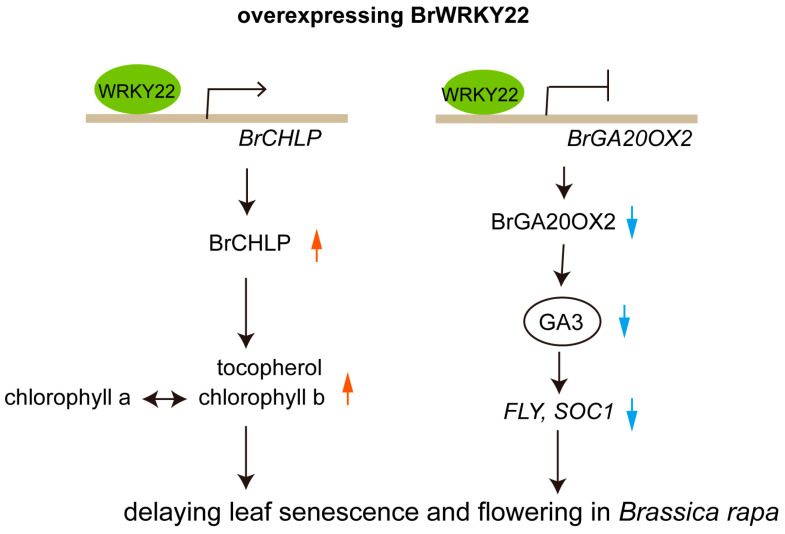
A schematic diagram of BrWRKY22 functions in *Brassica rapa*. Red arrowhead indicates increase, blue arrowhead indicates decrease.

As a large superfamily of plant transcription factors, WRKY proteins have undergone extensive functional analyses over the last two decades. The vast majority of this research has focused on the involvement of WRKY transcription factors in biotic and abiotic stress responses in plants. Several WRKY proteins have been shown to play a role in certain specific plant developmental processes, metabolism, and biosynthesis and regulation of hormonal signals [18,38,39,40,41,42]. The function of WRKY22 in *Arabidopsis* and in other species such as sweet oranges, lilies, tomatoes, and rice has been reported, and WRKY22 plays role in plant development, hormone biosynthesis, and in response to environmental stresses [43,44,45]. In this study, our findings revealed that BrWRKY22 directly controlled the expression of *BrGA20OX2* and *BrCHLP*, and overexpressing *BrWRKY22* decreased the GA3 content and increased chlorophyll b, leading to delayed flowering and leaf senescence *Brassica rapa* plants (Figure 8).

BrWRKY22 binds to the promoter region of BrCHLP and BrGA20OX2 and directly controls the expression of *BrGA20OX2* and *BrCHLP*, and overexpressing BrWRKY22 decreases the GA3 content and increases chlorophyll b, leading to a delayed flowering and leaf senescence *Brassica rapa* plants.

BrWRKY22 is closely related to other WRKY22 members across plant species, especially with the WRKY22 members of dicotyledonous plants by evolutionary analysis. The gene structure, protein structure, and conserved motifs in the BrWRKY22 protein showed that it is closely related to AtWRKY22, which affects dark-induced leaf senescence, as well as plant morphogenesis, and AtWRKY22 is involved in flower shape control but does not affect flowering time [30]. However, in *Brassica rapa* plants overexpressing *BrWRKY22*, flowering and leaf senescence were significantly delayed compared to the control plants, a contrasting phenotype observed in *AtWRKY22* overexpressing plants. This may be because, on the one hand, BrWRKY22 showed significant difference from *AtWRKY22* in the cis-acting element comparison analysis in the promoters of *BrWRKY22* and *AtWRKY22*. For example, the 3-AF1-binding site, MRE, and TCT motif and other light-responsive cis-acting elements are present only in the *BrWRKY22* promoter (Supplementary Table S1), which might change BrWRKY22’s sensitivity to light. On the other hand, the expression pattern for *BrWRKY22* at different stages of leaf development is consistent with their critical role as negative regulators of leaf growth, based on the stunted leaves and stems of the *BrWRKY22* overexpression plants. When *BrWRKY22* was overexpressed in *A. thaliana*, the entire transgenic plants were weaker, their leaves were curled, thinner and smaller, and their stems were significantly thinner and softer, indicating that the overexpression of *BrWRKY22* severely affects plant growth and development. Partial similar phenotypes have been reported in other species, for example, overexpression of *CsWRKY22* in sweet orange plants exhibited dwarf phenotypes that had wrinkled but thickened leaves because overexpression of *CsWRKY22* increased cell size in the spongy mesophyll [46]. Recent research has revealed that allelic variations in the promoter of WRKY22 enhance the environmental adaptation of *Arabidopsis thaliana* and *Brassica* due to the allelic mutation in the ARRs-binding region of WRKY22, but the W-box in its promoter region is conserved and upregulated by WRKY70 under both drought and humid conditions [47]. These results suggested that the evolutionary function variation of WRKY22 across the species results from the WRKY member and other transcription factor regulatory networks [48]. WRKY TFs have the ability to not only govern their own expression but also to engage in intercommunication with additional regulatory pathways, including those associated with plant hormone signaling. This enables the integration of various stress signals and the initiation of suitable gene expression responses. In general, the self-regulation of WRKY TFs constitutes a crucial process that facilitates the ability of the plants to effectively respond and adapt to challenging environmental conditions [49].

Monocarpic senescence determines crop harvest time, yield, and quality [50]. It has been found that early leaf senescence is always accompanied by early flowering, but delayed leaf senescence does not necessarily cause late flowering [5,6,7]. Natural leaf senescence has been observed in the late-flowering constants mutant, suggesting that leaf senescence may be independent from flowering [8]. At present, AtWRKY1 promotes flowering by directly repressing *FLC* expression and induces leaf senescence by activating SA biosynthesis genes. WRKY1 directly activates the genes involved in N assimilation and transport for remobilization from senescing leaves to seeds [29]. In addition, AtWRKY22, a dark-induced WRKY member, plays the role of accelerating leaf senescence and early flowering under dark induction conditions [30]. In this study, BrWRKY22 delays flowering through the indirect repression of *FLY* and *SOC1* expression mediated by low GA3 levels. It has been reported that GAs are required for almost all major developmental processes, from seed germination, stem elongation, and leaf expansion to flowering, through promoting cell division and elongation [13,51]. For example, the GA-deficient mutant *ga1-3*, which lacks the *GA1* gene encoding the first enzyme involved in GA biosynthesis, fails to flower under short days (SDs) [52], because, during SDs, low GA levels lead to an accumulation of DELLA proteins. DELLAs are involved in the regulation of *LFY* in the shoot apical meristem (SAM) [53,54]. In addition to LFY, GA also promotes the expression of *SOC1* [55].

BrWRKY22 directly activates the *BrCHLP* gene, which is involved in chlorophyll biosynthesis, keeping leaf stay green and delaying leaf senescence. It has been reported that the enzyme geranylgeranyl reductase (CHLP) catalyzes the reduction of geranylgeranyl diphosphate to phytol diphosphate, producing tocopherol and chlorophyll [56]. On the one hand, chlorophyll catabolism is preceded in the chloroplast structure and proteome during leaf senescence [57]. Tocopherol also plays a key role in plant responses to abiotic stress and leaf senescence. Specifically, it protects chloroplast membranes from photooxidation and provides a more optimal environment for photosynthetic machinery by scavenging reactive oxygen species (ROS) [58]. On the other hand, a link can be established between chlorophyll metabolites and hormones due to their biochemical origin and similar biological roles. Three branches of the methylerythritol phosphate pathway, which are involved in carotenoid (also ABA) biosynthesis, biosynthesis of the phytol side chain of chlorophylls, and diterpenoid (GA) biosynthesis, have been identified in many plants [59]. GGPP is not only used for carotenoid biosynthesis but also serves as a direct substrate for the biosynthesis of the phytol side chain of chlorophylls and of diterpenoids such as gibberellins. The allocation of GGPP between different downstream metabolic branches largely determines the biosynthetic capacity of each branch [59]. Thus, geranylgeranyl reductase (CHLP) catalyzes the reduction of geranylgeranyl diphosphate to phytol diphosphate and provides phytol for both chlorophyll (Chl) and tocopherol synthesis [60,61,62], maintaining the metabolic flux from the carotenoid (also ABA) biosynthesis pathway to biosynthesis of the phytol side chain of chlorophylls. This can explain that overexpressing *BrWRKY22* promotes chlorophyll b and tocopherol accumulation but declines the carotenoid and ABA contents, maintaining leaf stay green and delaying leaf senescence.

Conclusively, overexpression of BrWRKY22 resulted in stay green leaves, delayed flowering, and stunted plants, which were characterized by the severely inhibited growth of expanding leaves and elongating stems due to a lower GA3 content and higher chlorophyll b content. BrWRKY22 directly controlled the expression of *BrGA20OX2* and *BrCHLP*. We provided evidence that BrWRKY22 plays a role in plant growth and development. This research provides the basis for manipulating the *BrWRKY22* gene to control flowering and leaf senescence in *Brassica* plants.

## 4. Materials and Methods

### 4.1. BrWRKY22 Protein Sequences Retrieval

The amino acid sequences of the BrWRKY22 transcription factor, along with other monocots and dicot WRKY22 sequences, were downloaded from the National Center of Biotechnology Information (NCBI) (www.ncbi.nlm.nih.gov). Homologs of BrWRKY22 were chosen using the NCBI ‘Protein BLAST’ (https://blast.ncbi.nlm.nih.gov/Blast.cgi, accessed on 5 September 2023) online tool. GenBank accession numbers of the WRKY22 members are mentioned in the Supplementary Data.

### 4.2. Phylogenetics Analysis of the BrWRKY22 Protein

Protein sequences of BrWRKY22 and its homologs were aligned in the ‘Structural Alignment’ method using the multiple sequence alignment server TCOFFEE (http://tcoffee.crg.cat/, accessed on 5 September 2023) [63]. Maximum likelihood was used to construct phylogenetic trees using the MEGA7 program, and the confidence levels of the monophyletic groups were estimated using bootstrap analyses of 1000 replicates [64].

### 4.3. BrWRKY22 Gene Structure Analysis

BrWRKY22 CDS and genomic sequence were collected from NCBI (https://www.ncbi.nlm.nih.gov/) under accession numbers NM_001302033.1 and AENI01007266.1, respectively. The gene structure of BrWRKY22 was calculated from the gene structure display server (https://gsds.cbi.pku.edu.cn/).

### 4.4. Identification of Cis-Acting Elements in the Promoter Region of BrWRKY22

PlantCARE (http://bioinformatics.psb.ugent.be/webtools/plantcare/html/, accessed on 5 September 2023; v.2) online software was used to identify the cis-acting elements in the promoter region (1 kb upstream of the transcription start site) of BrWRKY22.

### 4.5. Plant Materials and Growth Condition

Chinese cabbage (*Brassica rapa*) cultivar “oil green 49” and *Nicotiana benthamiana* were selected as plant materials for phenotypic observation and subcellular localization, respectively. Plants were grown in a growth chamber at 22 °C with a 11-h/13-h dark/light photoperiod and 60% relative humidity. The light intensity of the chamber was adjusted to 100 μE/h.

For the development of *Brassica rapa* cultivar “oil green 49” transgenics, a growth chamber at 24 °C, a 16-h/8-h light/dark photoperiod, 60% relative humidity, and 100 μE/h light intensity was used.

The overexpressed BrWRKY22 in *Brassica rapa* were generated by *Agrobacterium*-mediated transformation and cultivation. As the basic and important step of transformation, the shoot regeneration procedures from the explants were established. The most efficient shoot regeneration system was achieved using cotyledon explants. After five days of planting (Figure 4A), those grown-up plantlets were cut from their hypocotyl region to obtain two different cotyledons from each plant. These cotyledons were grown in the pre-culture medium for three days (Figure 4B). The GV3101 strain was used for Agrobacterium tumefaciens at 10–15 min of infection and 2 days of co-culture (Figure 4C). After two days of the explant growth in the co-culture medium, those were transferred into the recovery medium (Figure 4C) for four days. The plants were kept in a screening medium until the emergence of a new plantlet. The screening medium was changed every week to ensure the required energy for callus and plantlet induction. A rooting medium was used to grow roots from new plantlets. Before transferring the plantlets into the rooting medium, they were kept in the shooting medium to obtain enough growth in the shoot (Figure 4G,H). The rooting and shooting mediums were changed every three weeks to ensure enough energy in the medium. The compositions of different mediums were used for the *Agrobacterium*-mediated gene transformation in *Brassica rapa* (Supplementary Table S2).

### 4.6. Yeast Two-Hybrid (Y2H) Assay

The full-length cDNA of *BrWRKY22* was cloned into plasmid vectors pGADT7 and pGBKT7 using DNA Ligation Kit verb 2.1 (TAKARA) to construct various bait and prey constructs. The primers for amplifying BrWRKY22 are listed in Supplementary Table S1. Then, using polyethylene glycol (PEG), different combinations of bait and prey constructs were co-transformed into yeast strain AH109, and yeast cells were grown on synthetic defined (SD) medium with -Leu/-Trp and selected in SD medium with -Leu/-Trp/-Ade and higher stringency SD/-Leu/-Trp/-His/Ade plates. After 3 days of incubation at 28 °C, the growth of each strain was measured. The transformants containing empty plasmids pGADT7 and pGBKT7 served as the negative control. Three biological replicates were performed for each combination in every growth assay.

### 4.7. Yeast One-Hybrid Assay

Yeast one-hybrid screening was performed using the MATCHMAKER One-Hybrid Library Construction Kit (Clontech, Mountain View, CA, USA). The full-length CDS of *BrWRKY22* was cloned into the pGADT7 vector. The promoter region-containing upstream region fragments (1800 bp) of *BrGA20OX2*, *BrGASA6*, *BrCHLP*, and *BrNYC1* were cloned into the pHIS2 reporter vector. Positive clones were identified by sequencing with AD sequencing primers. An empty pHIS2 reporter vector plus an appropriate concentration (50 mM) of 3-amino-1,2,4-triazole was used to inhibit HIS leakage.

### 4.8. Subcellular Localization of BrWRKY22 in Arabidopsis and Nicotiana benthamiana

BrWRKY22 subcellular localization was initially predicted by various subcellular localization prediction tools such as UniProtKB (https://www.uniprot.org/, accessed on 10 September 2023), Plant-mPLoc (http://www.csbio.sjtu.edu.cn/bioinf/plant/, accessed on 10 September 2023), LOCALIZER (http://localizer.csiro.au/ accessed on 10 September 2023), WoLF PSORT (https://wolfpsort.hgc.jp, accessed on 10 September 2023), and BaCelLo (http://gpcr.biocomp.unibo.it/bacello/pred.htm, accessed on 10 September 2023).

The open reading frame (ORF) of *BrWRKY22* (Bra037368) without the termination codon was cloned into the pPZP212-GFP-HA vector to generate the 35S:BrWRKY22-GFP construct, driven by the cauliflower mosaic virus 35S promoter. The 35S:BrWRKY22-GFP (Supplementary Figure S1) plasmid was extracted to obtain a plasmid concentration of at least 1 μg/μL. After that, epidermal strips of onion (*Allium cepa*) were placed in Murashige and Skoog (MS) medium. Both the 35S:BrWRKY22-GFP plasmid and the vector 35S:GFP (pPZP212-GFP-HA) were introduced transiently into onion epidermal cells by a helium-driven particle accelerator (PDS-1000; Bio-Rad, Hercules, CA, USA) with gold particles (1.0 mL) and a helium pressure of 1100 psi. After bombardment, the cells were incubated for 16 h at 22 °C in the dark. The nuclei were stained with 100 mg/mL of 49,6-diamidino-2-phenylindole (DAPI) in phosphate-buffered saline for 4 min [65] and observed under a confocal microscope.

Three-week-old *Nicotiana benthamiana* leaves were infiltrated with GV3101 plasmid harboring the 35S:BrWRKY22-GFP vector [66]. DAPI staining was performed and GFP fluorescence was detected using confocal microscopy 4 days post-infiltration (dpi).

### 4.9. Transformation of the pCBIM-Bra037368-Flag Vector into Arabidopsis thaliana

The *BrWRKY22* CDS region was amplified and inserted into the pCBIM vector to construct pCBIM-Bra037368 (BrWRKY22)-Flag. Restriction enzymes Xba1 and Kpn1 were used to prepare the complete pCBIM-Bra037368 (BrWRKY22)-Flag vector. The primers are mentioned in Supplementary Table S2.

To explore the role of WRKY22, we generated *A. thaliana* transgenic lines, harboring the pCBIM-BrWRKY22-Flag vector transformed into Arabidopsis ecotype Columbia using the floral dip method [67]. For the screening of the positive plants, the developed transgenic plants were screened for two generations on hygromycin-resistant plates. The *BrWRKY22* gene’s (harboring FLAG: pCBIM-BrWRKY22-Flag) successful transformation into A. thaliana transgenic lines was confirmed with the FLAG antibody through Western blotting. The phenotypic characteristics of the transgenic and WT plants were observed at different developmental stages.

### 4.10. Agrobacterium-Mediated Transformation of the OE Vector in Brassica rapa

*BrWRKY22* CDS was inserted into the empty pPZP212-Flag plasmid to construct pPZP212-BrWRKY22-Flag. The successfully developed constructs (35S:BrWRKY22-GFP; pCBIM- BrWRKY22-Flag vector; pPZP212-BrWRKY22-Flag) were transferred into Agrobacterium competent cell GV3101. To transfer the vector pPZP212-BrWRKY22-Flag into the *Brassica rapa* genome, different kinds of mediums (planting, pre-culture, co-culture, recovery, screening, shooting, and rooting mediums) were used accordingly. The compositions of all the mediums are given in Supplementary Table S3. The constructs were transformed into *Brassica rapa* explants using Agrobacterium-mediated transformation and overexpressing transgenic plants confirmed through PCR and qPCR (list of primers is mentioned in Supplementary Table S2).

### 4.11. SDS-PAGE and Western Blotting

Overexpressed plant lines were confirmed by performing Western blot analysis using the Sigma-Aldrich Western Blot Kit (St Louis, MO, USA). As the overexpression vector contained a FLAG antigen, a FLAG antibody was used to detect the band in Western blotting.

### 4.12. Real-Time PCR Analysis

RNA was extracted from the harvested samples using a ‘mini-RNA kit’ (Cowin Biosciences Co., Ltd., Taizhou, Jiangsu, China). Ultra SYBRmixture reagent (CWBIO, Taizhou, China) was used to perform qRT-PCR on a CFXConnect real-time PCR machine (BIO-RAD, Hercules, CA, USA). Three biological replicates of each sample (each having three technical repeats) were used for this experiment. The glyceraldehyde 3-phosphate dehydrogenase (GAPDH) reference gene was used for the internal standardization of the quantitative real-time PCR data. Livak and Schmittgen’s data analysis method was followed to calculate the relative expression [68]. A list of the primers used for RT-qPCR is enlisted in Supplementary Table S2.

### 4.13. Measurement of Chlorophyll and Hormones

Leaves of 3-week-old overexpressing lines and wild-type *Brassica rapa* plants were collected, and the contents of the hormones were measured by HPLC [69,70]. Briefly, approximately 100 mg of freeze-dried leaves were homogenized with 1 mL of a methanol–dimethyl sulfoxide mix (1:1, *v*/*v*) at 40 °C for 30 min with agitation (190 rpm) in darkness. A final volume of 10 L of homogenate was used for injection into the HPLC instrument. The following reagents were purchased: standards GA3 and ABA from Sigma-Aldrich (St. Louis, MO, USA). All standards were prepared with methanol–dimethyl sulfoxide (1:1, *v*/*v*) and stored at −20 °C before use.

Fresh leaf tissues (~200 mg) from the overexpressing lines and WT were immersed in 10 mL of extract solution for 48 h in the dark. The absorbance of the supernatant was measured with a dual-beam ultraviolet spectrophotometer (TU1901, Beijing puxi general instrument company, Beijing, China) at 470, 649, and 665 nm. The Chl a and Chl b contents and the total carotenoids contents were calculated according to the method described by Ren et al. (2017) [69]. The data are expressed as the mean ± SDs of three biological replicates.

### 4.14. Dual-Luciferase Assay

The dual-luciferase experiment was conducted using the method of Rodrigquez et al. (2004) [71], with slight changes. Agrobacterium GV3101 (pSoupp19) competent cells were transfected with 35S:BrWRKY22-GFP overexpression vector (the effector); the PBrCHLP:LUC, PBrNYC1:LUC, PBrGASA6:LUC, and PBrGA20OX2:LUC vectors (the reporters); and the 35S:GFP empty vector (as negative controls for the effector). The Agrobacterium resuspending solution containing various combinations of effector and reporters was mixed in an equal ratio (1:1) and used for tobacco injection. The leaves were injected with various combination vectors. The injected tobacco plants were cultured in the dark for 12–24 h. After 2–3 d, the GFP fluorescence signal was visualized under a laser confocal microscope (Leica SP8). The excitation and emission wavelengths of GFP (488 nm, 500–560 nm), DAPI (405 nm, 420–470 nm), and chloroplast (488 nm, 650–750 nm) were measured, respectively. All the captured images were processed using ImageJ (http://rsb.info.nih.gov/ij/Fiji, accessed on 10 September 2023).

The luciferase activity of each sample was qualitatively analyzed using an in vivo plant imaging system (Immunization Center of Fujian Agriculture and Forestry University). Subsequently, the relative luciferase activity was quantified with a microplate reader [72]. The ratio of firefly to Renilla luciferase activity (LUC/REN) was calculated as the relative luciferase activity.

### 4.15. Statistical Analysis

The data in all figures were determined by at least three biological replicates, with each replicate consisting of three samples. To evaluate the statistical significance, one-way ANOVA was used, followed by the multiple factors Tukey’s HSD test or Student’s *t*-test. Asterisks denote statistical significance, with *p* < 0.05 indicating a substantial difference. All of them were done with GraphPad Prism software, version 8 (GraphPad Software, San Diego, CA, USA).

## Data Availability

All data in this study are provided in the figures and Supplementary Information.

## Supplementary Materials

The following supporting information can be downloaded at https://www.mdpi.com/article/10.3390/plants14111658/s1: Supplementary Figure S1: Gene structure of *BrWRKY22*. Supplementary Figure S2: Selection of OE BrWRKY22 *Arabidopsis thaliana* plant lines in the antibiotic medium. Supplementary Figure S3: Confirmation and selection of OE BrWRKY22 *Arabidopsis thaliana* plant lines based on the protein expression. Supplementary Table S1: Comparative analysis of cis-acting elements in the promoter region of BrWRKY22 and AtWRKY22. Supplementary Table S2: Comparison of different mediums used for Agrobacterium-mediated gene transformation in *Brassica rapa*. Supplementary Table S3: The list of primer sequences in this study.
