# Supplementary material for: Overexpressing BrWRKY22 Delays Flowering and Leaf Senescence via Inhibition of GA Biosynthesis in Brassica rapa
Source: Plants (Basel). 2025 May 29;14(11):1658. doi: 10.3390/plants14111658 (PMC12157233; doi:10.3390/plants14111658)
Supplement: Supplementary file 1 [file plants-14-01658-s001.zip › plants-3632624-supplementary.pdf]

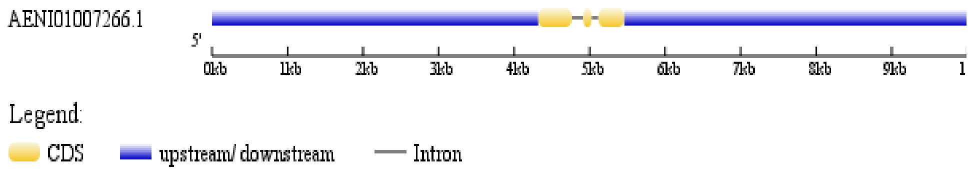

Supplementary Figure S1: Gene structure of *BrWRKY22*. Yellow color CDS (exon), Blue color shows UTR (untranslated regions) while the normal line represents introns.

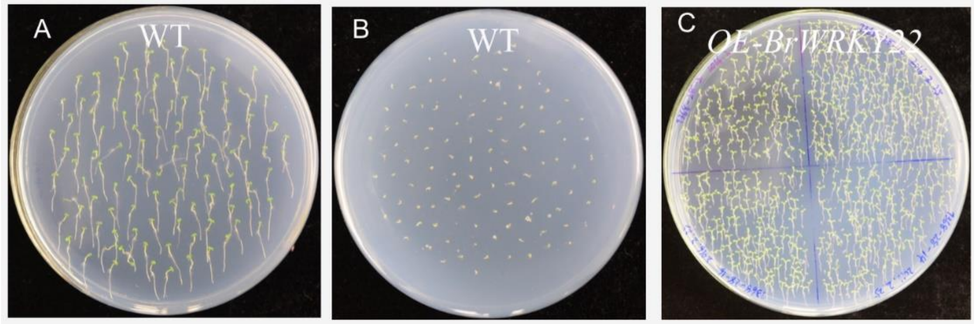

Supplementary Figure S2: Selection of OE BrWRKY22 Arabidopsis plant lines in the antibiotic medium. A) Screening effect of wild type seeds in the plate without antibiotic showing these may grow easily in hygromycin less medium. B) Screening effect of WT seeds in the plate with hygromycin. C) Screening effect of the transgenic seeds in the resistant plate showing the transformation of pCBIM-Bra037368 (BrWRKY22)-Flag vector into Arabidopsis thaliana and Brassica rapa was successfully performed.

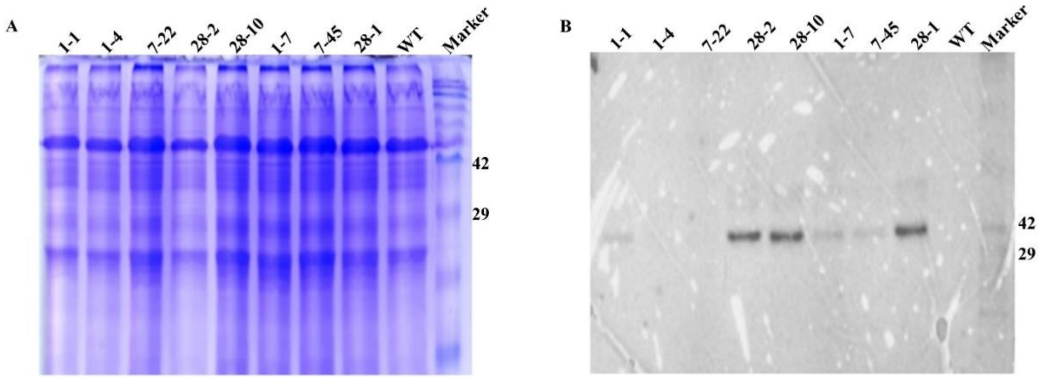

Supplementary Figure S3: Confirmation and selection of *OE BrWRKY22 Arabidopsis thaliana* plant lines based the protein expression. A) SDS-PAGE assay of leaves total-proteins showing good protein quality in each plant lines. Lane WT indicates the wild type; Other lanes with different numbers indicate the *OE-BrWRKY22 Arabidopsis thaliana* plant lines. B) In western blot analysis 28-2, 28-10, and 28-1 plant lines showed bright bands against FLAG antibody. Indicating these are potential *BrWRKY22* over-expression plant lines.

Supplementary Table S1: Comparative analysis of *cis*-acting elements in the promoter region of *BrWRKY*22 and *AtWRKY*22.

Element	Function	Number of element		Class
		Br	***At***	
Box 4	Part of a conserved DNA module	1	1	Light responsive elements
3-AF1 binding site	Light responsive element	1		
MRE	MYB binding site	1		
TCT-motif	Part of a light responsive element	1		
GT1-motif	Light responsive element		2	
CGTCA-motif	MeJA-responsiveness		3	Hormone-responsive elements
SARE	salicylic acid responsiveness		1	
TCA-element	salicylic acid responsiveness		1	
TGACG-motif	MeJA-responsiveness		3	
ARE	essential for anaerobic induction	3	1	Elements related to anaerobic induction
TATA		1	1	Basic Elements
AT~TATA-box		2	2	
TATA-box	Core promoter element	35	28	
CAAT-box	Common *cis*-acting element	18	20	
MYB		2	4	In both genes
MYB-like		2	4	
MYC		3	2	
Myb		1		
Unnamed__4		7	4	
A-box	*Cis*-acting regulatory element	1		Only in *BrWRKY22*
AT-rich element	Binding site of AT-rich DNA binding protein (ATBP-1)	1		
CCGTCC motif		1		
ERE		2		
CCAAT-box	MYBHv1 binding site		1	Only in *AtWRKY22*

Supplementary Table S2: Comparison of different medium used for *Agrobacterium*-mediated gene transformation in *Brassica rapa*.

**Medium Name**	**MS powder**	**Sucrose**	**Agar**	**6 BAP**	**NAA**	**Ampicillin**	**Kanamycin**	
Planting	2.215 g	10 g	1.6 g					
Pre-culture	2.215 g	10 g	1.6 g	2 mgL^{-1}	0.45 mgL^{-1}			
Co-culture	2.215 g	10 g	1.6 g	2 mgL^{-1}	0.45 mgL^{-1}			
Recovery	2.215 g	10 g	1.6 g	2 mgL^{-1}	0.45 mgL^{-1}	300 mgL^{-1}		
Screening	2.215 g	10 g	1.6 g	2 mgL^{-1}	0.45 mgL^{-1}	300 mgL^{-1}	5 mgL^{-1}	
Shooting	2.215 g	10 g	1.6 g	2 mgL^{-1}	0.45 mgL^{-1}	300 mgL^{-1}	5 mgL^{-1}	
Rooting	2.215 g	10 g	1.6 g		0.6 mg/L	300 mgL^{-1}	5 mgL^{-1}	

The final volume of each medium was adjusted to 500ml using RO water. M519 MS powder and Gelzan agar were used. Both of these chemicals are produced from the Phytotechnology laboratory, US.

Supplementary Table S3: The list of primer sequences in this study.

Primer Name	Sequence (5'-3')
**Subcloning**	
BrWRKY22-BamH I-CL-FP	gcccggatccCTGAGAAACCAAGTCAAATGGCCGACG
BrWRKY22-Spe I-CL-RP	TCATactagtTATTCCACCGCTAGCTGTGGCAGC
B. rapa-GAPDH-QPCR-FP	CCACTAACTGCCTTGCTCCACTT
B. rapa-GAPDH-QPCR-RP	GCGGCTCTTCCACCTCTCCAGT
BrWRKY22-Sem/Q-PCR-FP	ACTAGCAAACCCACAGGAGC
BrWRKY22-Sem/Q-PCR-RP	GGAGACTTAGTCGTCGGCTG
BrWRKY22-Nco I-TA-FP	ATGGCCATGGATATGGCCGACGATTGGGATC
BrWRKY22-Sac I-TA-RP	CGACGAGCTCGTATTCCACCGCTAGCTGTGGCAGC
BrWRKY22-BamHI-AD-F	TACGGGATCCATATGGCCGACGATTGGGATC
BrWRKY22-CBIM-Spe I-FP	tctactagtCTGAGAAACCAAGTCAAATGGCCGACG
BrWRKY22-CBIM-Kpn I-RP	GTAggtaccTATTCCACCGCTAGCTGTGGCAGC
BraWRKY22-F	CGACGACTAAGTCTCCGACG
NOST-R	TAATCATCGCAAGACCGGCA
**RT-qPCR**	
BrWRKY22-FP	AAGTGTGGAGGATCAATCAG
BrWRKY22-RP	ATGGGCTTGTCACTTTCTTA
BrNYC1-FP	GATGTCTGGAACACCGCCTC
BrNYC1-RP	TTCTCACGCTTTCTCCGTCTTT
BrCHLP-FP	GAAACTACCAACGGCGACAAGAAG
BrCHLP-RP	CCGGTGTCCTCACGTCCAGA
BrSGR1-FP	CAAAGCTCCCAAGAACTTACACTC
BrSGR1-RP	AGATTCTTGTAGCTCAGGGTAACT
BrGA20OX2-FP	TCACCATCAYYGGMTCRCCTC
BrGA20OX2-RP	AGATCATCGCGAAWWACYTGTA
BrGASA6-FP	TGGAGGGGAATGCACTAGGA
BrGASA6-RP	TGTTTGTTGCCGTAAGTGCC
BrLFY-FP	CGAACAGTGTCGCGAGTTTC
BrLFY-RP	GTAGTGTCGCATCTTCGGCT
BrGAPDH-FP	CAGGTTTGGAATTGTCGAGG
BrGAPDH-RP	GAGCTGTGGAAGCACCTTTC
BrSOC1-FP	CACATCCTCTGTTTTGTTCACGAC
BrSOC1-RP	AAGAGTTTGTTCCTGGAGTTGTTCC

Supplementary Data: GenBank accession numbers of different WRKY22 members from different species: NP_001288962.1, OAO99945.1, AJF11720.1, RDX97488.1, AAO23325.1, NP_001147816.1, ABI13388.1, ACQ76802.1, DAA05087.1, ACD80376.1, KHG04290.1, XP_013608996.1, XP_018453027.1, XP_002872898.1, XP_006396285.1, XP_010422786.1, XP_010544418.1, XP_012490022.1, XP_022770504.1, XP_007051705.2, XP_021683420.1, XP_021613059.1, AZQ19342.1, XP_002511920.1, XP_021807936.1, XP_006375351.1, XP_009341117.1, XP_022871142.1, XP_011015965.1, XP_007218198.1, XP_008232978.1, XP_011092990.1, XP_027336872.1, XP_021887800.1, XP_010092573.1, XP_022924275.1, KHN18817.1, XP_015061786.1, XP_022980798.1, XP_009768958.1, and XP_024175977.1.
